# Smoking habits and factors that influence them among school students from rural areas of Romania

**DOI:** 10.18332/tid/214406

**Published:** 2026-02-14

**Authors:** Tania E. Tudor, Hein de Vries, Lucia M. Lotrean

**Affiliations:** 1 Department of Community Medicine, Iuliu Hatieganu University of Medicine and Pharmacy, Cluj Napoca, Romania; 2 Research Center in Preventive Medicine, Health Promotion and Sustainable Development, Iuliu Hatieganu University of Medicine and Pharmacy, Cluj Napoca, Romania; 3 Department of Health Promotion, Maastricht University, Maastricht, Netherlands

**Keywords:** smoking behaviors, adolescents, rural areas, Romania

## Abstract

**INTRODUCTION:**

The aim of the study was to evaluate smoking habits and the factors influencing them among school students in rural areas of Romania.

**METHODS:**

The study sample included 748 school students aged 13–14 years from twenty-four schools from rural areas situated in two counties of Romania. The cross-sectional study was conducted in 2019 among participating students using confidential questionnaires to assess smoking-related behaviors and the factors that influence them.

**RESULTS:**

Our findings revealed that 15.9 % of the sample were current smokers (no statistically significant differences were noticed between boys and girls). Smoking prevalence was higher among students without household goods such as a phone or a computer, compared to those who owned them (28.4% vs 13.8%, p<0.001, and 25.2% vs 14%, p<0.01, respectively). Students with lower school results also had higher smoking rates than those with better results (21.1% vs 8.1%, p<0.001). Adolescents who consumed alcohol monthly had higher smoking prevalence compared to those who did not (41.2% vs 13.4%, p<0.001). Engaging in fights monthly (34.6% vs 15%, p<0.001), vandalism (44.1% vs 14.3%, p<0.001), and stealing monthly (50% vs 15.2%, p<0.01) were also linked to higher smoking prevalence compared to not having these behaviors. Spending most spare time in bars/discos was associated with higher smoking prevalence (60% vs 14.9%, p<0.001). Practicing sports was correlated with lower smoking prevalence than not practicing sports (12.7% vs 16.4%, p<0.05). Smokers were more likely to perceive the benefits of smoking, whereas non-smokers were significantly more convinced about several disadvantages of smoking. Additionally, the study found an increased perceived behavior and pressure to smoke from parents, siblings, friends, best friends, and peers in the same school year among smokers. Smokers had lower self-efficacy to refrain from smoking in different situations. Intention to smoke in the next year was higher among smokers.

**CONCLUSIONS:**

The results have implications for the development of health promotion activities for smoking prevention among Romanian adolescents from rural areas.

## INTRODUCTION

The global impact of the tobacco epidemic is overwhelming, representing one of the most significant challenges to public health. According to the World Health Organization (WHO), tobacco kills more than eight million people each year (more than seven million due to direct tobacco consumption, 1.3 million due to passive smoking). Also, over 80% of the world’s 1.3 billion tobacco users live in low- and middle-income countries^1,2^.

In Romania, according to the Global Youth Tobacco Survey (GYTS), conducted in 2017 among students, aged 13–15 years, by the National Institute of Public Health, 14.6% were current tobacco product users, and 8.6% were current cigarette smokers; 28.0% of ever cigarette smokers first tried a cigarette before the age of 10 years (Global Youth Tobacco Survey 2017 - Romania). These statistics are worrying, especially because of the observation that starting smoking at an early age can be a predictor of nicotine dependence in adulthood. Even infrequent smoking in adolescence can lead to smoking addiction, increasing the risks of premature death and diseases^3-5^.

A Polish study showed that smoking prevalence was higher among adolescents, aged 15–19 years, living in rural areas (30.57%) than those who were living in urban areas (25.61%)^6^. In Romania, there are limited data regarding smoking behavior and associated factors among adolescents from rural areas.

The Integrated Model for explaining motivational and behavioral change (I-Change), developed by de Vries^7^, is a theoretical framework that integrates elements from several theories of behavior change, which is used to understand and predict health-related behaviors and to guide the development of interventions that lead to behavior changes. The I-Change Model, like other social-cognitive health behavior models, assumes that an important determinant of behavior is behavioral intention, which is in turn influenced by three proximal factors: individuals’ overall evaluations of the behavior (attitude), their beliefs about the beliefs and behaviors of significant others (social influence), and the control that they perceive themselves to have over performing a behavior (self-efficacy). Distal factors such as biological, psychological, behavioral, and socio-cultural characteristics are assumed to influence behavior via these proximal factors^8-10^.

The purpose of this study is to evaluate the prevalence of smoking among Romanian adolescents in rural areas and to identify factors associated with smoking. The study is guided by the I-change model and focuses on sociodemographic, health-related behavior, and motivational determinants of smoking (attitudes, social influences, self-efficacy, and intention). This approach can provide valuable insights for developing appropriate smoking prevention programs that address the specific needs and challenges of this population.

## METHODS

### Study sample and procedure for data collection

Within this cross-sectional study, the schools involved in the study were situated in rural areas from two counties in Romania: Cluj County (North-Western side) and Arad County (West side). Principals of twenty-six schools located in Arad County and Cluj County were asked to participate with the school they lead in a smoking prevention program. The schools were randomly selected from the secondary schools’ public list on the website of the school inspectorate of each county. Thirteen out of eighty-seven gymnasium schools located in Arad County, and thirteen out of ninety-two gymnasium schools located in Cluj County were selected. Twenty-four out of twenty-six school principals agreed to participate in the project and provided the number of students from the 7th and 8th grades who could participate.

In autumn 2019, confidential questionnaires were distributed to participating middle schoolers; the median time of completing the questionnaire was 50 minutes. Based on the total of students in 7th and 8th grade from Arad and Cluj County (approx. 5500 students), power calculations were run, applying a confidence level of 95% and error margins of 3%^11^. Based on previous studies, an estimated smoking prevalence of 15% was considered. The recommended sample size was 496^11^.

The questionnaires were filled in by 760 students out of 825 who recieved parental consent, because 65 were absent on the days of assessment. Of those 760, 12 students were excluded because their questionnaires were incomplete, leaving 748 (50.6% girls, 49.4% boys).

The study was approved by the Ethics Committee of Iuliu Hatieganu Medicine and Pharmacy University, Cluj Napoca, Romania (Approval number: 193; Date: 19 April 2018). Parental informed consent was obtained for students’participation in the study; 825 out of 1172 parents had approved their children’s participation.

### Instruments for data collection

The questionnaire used in this study was based on questionnaires previously used and tested in several studies from Romania and other European countries^12-15^. It included several sections investigating health risk behavior among participating middle-schoolers, with a special focus on smoking behavior and e-cigarette use. The present study includes information collected through the confidential questionnaires concerning sociodemographic characteristics, involvement in several health risk behaviors, smoking behavior, motivational factors regarding smoking (attitudes, social influences, self-efficacy expectations, intention). The participants received the questionnaire with words. All the numeric values were added to the database after all the data had been collected.

The first page contained information about the rules of filling in the questionnaires, voluntary participation, and assured students’ confidentiality. The teachers were in the classroom during the data collection, but they did not intervene to ensure the confidentiality of the answers. Completed questionnaires were collected by the research team in closed envelopes with students’ names on them. Participants who did not want to participate did not return the questionnaires. A confidential approach was used to develop a longitudinal study over one year concerning these issues. Demographic variables assessed by questionnaire were age (13–14 years), gender (0=boys, 1=girls), religious background (none, orthodox, catholic, protestant, other), family status (living with both parents: no=0/yes=1), and several other characteristics. Data about household goods (private property, car, computer, etc.) were also collected to obtain information about the socioeconomic status. School performance was also assessed (0=in the bottom two-thirds of their class, 1=in the top third of their class), but also places where adolescents spent most of their spare time (at home, friend’s house, in parks, on the street, in shops, discos/bars, sport clubs).

The risk-behavior (use of alcohol, gambling, fighting, vandalism, stealing monthly) was also evaluated with eight items on a five-point scale; variables with two possibilities of answer were created (0=less than once a month, 1=at least once a month).

Smoking behavior was assessed by a combination of two questions. In the first question, middle-schoolers were asked to choose a statement that best described their smoking behavior (e.g. ‘at least once a day/week/month’, ‘less than once a month/sometimes’, ‘I smoked sometimes, but I don’t smoke anymore’/‘I never smoked’). The second question assessed lifetime cigarette consumption (less than one hundred, more than one hundred, none). Based on those questions, participants were categorized into two categories: current smokers and non-smokers. According to the Centers for Disease Control and Prevention, a current smoker is defined as a person who reported smoking more than 100 cigarettes during their lifetime and who, at the time they participated in this survey, reported smoking. In the definition of the smoker, we did not include other combustible tobacco products (e.g. cigars, cigarillos, hookah)^16^. Current smokers were considered those who reported smoking weekly or who smoked more than 100 cigarettes during their lifetime. The remaining participants were classified as non-smokers.

Attitudes were measured on a seven-point scale using twelve items. Two scales were created based on the outcomes: pros and cons of smoking. The pro-smoking scale was created by nine questions (Cronbach’s α=0.774) while the cons-smoking scale was created by six questions (Cronbach’s α=0.737). Pro-smoking arguments referred to positive aspects of smoking (e.g. ‘it helps me calm my nerves’). The arguments against smoking refer to negative outcomes of smoking (e.g. ‘It is bad for my health’). Answering categories ranged from ‘I totally disagree’ (-3) to ‘I totally agree’ (3).

The perceived social influences included three groups of questions: social norms, social modeling, and social pressure from family members (father, mother, brother, sister), and peers (best friend, friends, and people in the same school year).

Adolescents’ perception of social norms regarding smoking was evaluated through seven questions rated on a seven-point scale measuring adolescents’ perception about the belief that parents, siblings, and friends think they should smoke or not, e.g. ‘My mother thinks I definitely should smoke’ (3) and ‘Definitely should not smoke’ (-3). Three meaningful factors were revealed, based on which three scales were constructed: for parents (sum score of mother and father, Cronbach’s α=0.775), siblings (sum score of brother and sister, Cronbach’s α=0.516), and peers (sum score of friends, best friend, and peer in the same school year, Cronbach’s α=0.785).

Social modeling assesses students’ perception of smoking within their social circles. Perceived behaviors of parents, siblings, and best friend were measured on a two-point scale (0=no, 1=yes), while for friends and people in the same school year, a five-point scale was used (from ‘almost everybody’ to ‘almost nobody’). The perceived behavior was examined individually for each student because behaviors are not one-dimensional and cannot be simplified in a scale.

Social pressure assessed the pressure of smoking that students feel from different people and was measured by eight questions of a five-point scale ranging from ‘never’ to ‘very often’. For example: ‘Have you ever felt pressure to smoke from your mother?’. Response options ranged from ‘very often’ (4) to ‘never’ (0). Factor analysis revealed three stable factors. Three scales were created: pressure from parents (sum score of mother and father, Cronbach’s α=0.819), pressure from siblings (sum score of sister and brother, Cronbach’s α=0.746), and pressure from peers (sum score of best friend, friends, and people in the same school year, Cronbach’s α=0.804).

Self-efficacy was measured by twelve questions on a seven-point scale and assessed attitudes regarding refraining from cigarettes in different situations (e.g. ‘when other people smoke’/‘when I am sad’). The answer options ranged from ‘I am sure I won’t smoke’ (3) to ‘I am sure I will smoke (-3). Factor analysis revealed one factor for self-efficacy (Cronbach’s α=0.971).

Intention to smoke was measured by one question on a seven-point scale, which evaluated the intention to smoke in the next year. The response possibilities were from ‘definitely yes’ (3) to ‘definitely no (-3)’.

Only for smokers, the methods of quitting smoking were evaluated by eight questions on a five-point scale, and they assessed what they would do to quit smoking, e.g. ‘I would inform my friends’. The response possibilities were from ‘I totally agree’ (2) to ‘I totally disagree’ (-2).

### Analysis

Chi-squared analysis revealed differences in smoking prevalence among students with different sociodemographic backgrounds. The Shapiro-Wilk test for normality was performed, and because the distribution was not normal (p<0.05), the Mann-Whitney U test was used to assess the differences between smokers and non-smokers concerning their attitudes, social influences, self-efficacy, beliefs, and intention to smoke in the next year. Item scores were used to gain detailed insights into the specific items that make a distinction between smokers and non-smokers.

To examine the correlation between smoking and the constructs of the I-Change Model assessed in our study, the scales were used as variables in a logistic regression analysis. Binary logistic regression analysis with the forward conditional method was used. The multivariate model was built according to the I-Change model. The dependent variable was smoking behavior. The independent variables included in the analysis in Model 1 were demographic variables [gender, living with both parents, ownership of a personal mobile phone, family ownership of a private property (apartment/house), phone, mobile phone, car, computer, TV]. In Model 2, risk behavior (use of alcohol at least once a month, gambling, fighting, destroying/stealing other people’s things at least once a month), school achievement, and the most frequented places in their free time (at home, friend’s home, bars/discos, parks, practicing sports) were included. In Model 3 were included the scales regarding attitudes, social norms, social pressure, social modeling, and self-efficacy items; while intention to smoke in the next year was included in Model 4.

Data analysis was done with IBM SPSS Statistics version 26.0 (IBM Corporation, Armonk, NY, USA). Significant results are reported as p<0.05.

## RESULTS

### Characteristics of the sample

The distribution by gender among the whole sample was 50.6% girls and 49.4% boys; 20.2% of the students belonged to disrupted families, and 97.7% declared that they belong to a religion (73.5% orthodox orientated).

Regarding the household goods, the results showed that more than 80% of participants’ families had their private property, refrigerator, own mobile phone, television, family phone, computer, and car; 60.3% declared that they were in the bottom two-thirds of their class in school performance in the last year.

Alcohol consumption at least once a month was reported by 9.1% of the subjects; 3.5% declared that they played gambling games at least once a month while 5.3% declared that they were fighting at school at least once a month; 4.5% destroyed other people’s things at least once a month, while 1.6% declared that they stole from others at least once a month.

Of the whole sample, 15.9% were current smokers, with no significant differences based on gender; 43.7% of the current smokers declared that they were daily smokers. The number of cigarettes smoked per week was <20 for 78.2% of the current smokers, 9.2% smoked 21–40 cigarettes/week, 5% declared that they smoked 41–60 cigarettes, while 7.6% declared that they smoked >60 cigarettes/week.

Concerning non-smokers, 78.2% were never smokers (they never smoked, not even tried), 5.6% were considered experimental smokers (smoked occasionally but not every week, and they have smoked less than one hundred cigarettes during their lifetime), and 16.2% were quitters (they stopped smoking).

### Differences between smokers and non-smokers regarding sociodemographic and health-related behaviors

Table 1 shows higher smoking frequency among those with lower socio-economic level and lower academic achievement, but also among students who declared risk behaviors. Students spending free time at home or practicing sports had a lower prevalence of smoking.

**Table 1:** Prevalence of smoking in subjects with different sociodemographic backgrounds and several health risk behaviors, Arad and Cluj Counties, Romania, 2019 (N=748)

*Characteristics*	*Smoking* *prevalence* *%*	p*
**Gender**		
Boys	16.2	0.852
Girls	15.7	
**Disrupted families**		
No	19.1	0.263
Yes	15.1	
**Household phone**		
No	28.4	0.000
Yes	13.8	
**Household computer**		
No	25.2	0.001
Yes	14.0	
**Private property** (apartment/ house)		
No	13.9	0.637
Yes	15.7	
**School achievement last year**		
In the top third	8.1	0.000
In the bottom two-thirds	21.1	
**Using alcohol at least once a month**		
No	13.4	0.00
Yes	41.2	
**Gambling at least once a month**		
No	15.0	0.07
Yes	34.6	
**Fighting at least once a month**		
No	14.3	0.000
Yes	45.0	
**Destroy other people's things at least once a month**		
No	14.3	0.000
Yes	44.1	
**Stealing things from others at least once a month**		
No	15.2	0.001
Yes	50.0	
**Spending most of the free time at home**		
No	34.4	0.000
Yes	14.3	
**Spending most of the free time in the park/on the street**		
No	13.8	0.032
Yes	17.2	
**Spending most of the free time in bars/ discos/parties**		
No	14.9	0.000
Yes	60.0	
**Spending most of the free time practicing sports**		
No	16.4	0.041
Yes	12.7	
**Spending most of the free time in a friend’s house**		
No	15.0	0.527
Yes	16.7	

* Chi-squared test.

### Differences between smokers and non-smokers regarding attitudes

Table 2 shows that smokers were more convinced about the benefits of smoking, while non-smokers were more aware of the disadvantages of this habit. However, smokers are also aware of the negative effects of smoking on their health, and they perceive smoking behavior as being wrong.

**Table 2:** Differences between smokers and non-smokers regarding attitudes, Arad and Cluj Counties, Romania, 2019

*Items*	*Non-smokers* *(N=629) Mean (SD)*	*Smokers* *(N=119) Mean (SD)*	p*
**Pros**			
Smoking is attractive	-1.29 (1.610)	-0.04 (1.734)	0.000
Smoking is pleasant	-1.39 (1.428)	-0.13 (1.745)	0.000
It helps to calm my nerves	-0.36 (1.107)	-0.26 (1.504)	0.000
I feel more mature	-0.80 (1.202)	-0.59 (1.399)	0.256
I feel more confident	-0.10 (1.102)	0.75 (1.360)	0.000
It will make me feel relaxed	0.01 (1.028)	0.95 (1.466)	0.000
It helps me to be slim	-0.41 (0.977)	-0.26 (1.182)	0.156
It is easier to be part of the crowd	-0.03 (1.425)	0.67 (1.541)	0.000
My friends will pay me more attention	-0.21 (1.416)	0.38 (1.334)	0.000
**Cons**			
It is bad for my health	1.49 (0.962)	1.00 (1.105)	0.000
It is stupid of me	1.17 (1.167)	0.55 (1.477)	0.000
I consider my behavior to be wrong	2.30 (1.126)	1.35 (1.549)	0.000
If I get sick, I will be sorry that I ever started	1.44 (1.047)	1.14 (1.392)	0.003
It tastes horrible	1.51 (1.556)	0.48 (1.808)	0.000
I believe it to be unfriendly	1.06 (1.463)	0.46 (1.437)	0.000

* Mann-Whitney U test.

### Differences between smokers and non-smokers regarding social influences

Table 3 shows that smokers perceive lower social norms against smoking, feel more pressure to smoke from parents, siblings, as well as friends and peers in the same school year. With regard to smoking behavior, it was more frequent among mothers, siblings, and the best friends of smokers, while no significant difference was found between the two groups with regard to smoking among fathers.

**Table 3:** Differences between smokers and non-smokers regarding social influences, Arad and Cluj Counties, Romania, 2019

	*Non-smokers* *(N=629) Mean (SD)*	*Smokers* *(N=119) Mean (SD)*	p*
**Social norms**			
Mother	-2.48 (0.947)	-1.95 (1.268)	0.000
Father	-2.39 (1.037)	-1.71 (1.427)	0.000
Brother(s)	-1.49 (1.369)	-1.08 (1.555)	0.015
Sister(s)	-1.41 (1.362)	-1.08 (1.544)	0.051
Friends	-1.64 (1.439)	-0.95 (1.677)	0.000
Best friend	-2.13 (1.169)	-1.10 (1.792)	0.000
People in the same school year	-1.72 (1.335)	-1.29 (1.508)	0.004
**Perceived smoking behavior**			
Mother	0.40 (0.491)	0.52 (0.502)	0.016
Father	0.45 (0.498)	0.51 (0.502)	0.221
Brother(s)	0.16 (0.446)	0.22 (0.415)	0.048
Sister(s)	0.09 (0.285)	0.15 (0.360)	0.037
Best friend	0.14 (0.347)	0.57 (0.497)	0.000
**Social pressure**			
Mother	0.09 (0.509)	0.64 (1.287)	0.000
Father	0.09 (0.485)	0.63 (1.248)	0.000
Brother(s)	0.13 (0.668)	0.55 (1.247)	0.000
Sister(s)	0.14 (0.741)	0.44 (1.154)	0.000
Friends	0.45 (0.858)	1.49 (1.395)	0.000
Best friend	0.15 (0.553)	1.33 (1.496)	0.000
People in the same school year	0.30 (0.717)	1.03 (1.298)	0.000

* Mann-Whitney U test.

### Differences between smokers and non-smokers regarding self-efficacy and intention

Table 4 shows that non-smokers exhibited higher levels of confidence in their ability to resist smoking, while smokers expressed lower self-efficacy expectations regarding not smoking, particularly in the company of smoker friends or when they are upset, depressed, or nervous. Smoking was related to positive intention to smoke in the next year. Supplementary file Table 1 indicates the analysis restricted only to smokers, and shows the respondents’ method by which they would quit smoking. The majority would inform friends, while fixing a date to quit is the least chosen quitting strategy in our study.

**Table 4:** Differences between smokers and non-smokers regarding self-efficacy and intention, Arad and Cluj Counties, Romania, 2019

*Items*	*Non-smokers* *(N=629) Mean (SD)*	*Smokers* *(N=119) Mean (SD)*	p*
**Situation**			
When with people who smoke	2.33 (1.156)	0.33 (1.979)	0.000
When with friends who smoke	2.32 (1.148)	0.09 (2.013)	0.000
When you are offered a cigarette	2.37(1.117)	0.31 (2.012)	0.000
When friends offer you a cigarette	2.34 (1.142)	0.22 (2.022)	0.000
When you feel upset	2.37 (1.172)	-0.20 (2.081)	0.000
When you feel depressed	2.35 (1.179)	-0.14 (2.156)	0.000
When you feel nervous	2.38 (1.187)	-0.26 (2.238)	0.000
When you are worried	2.41 (1.120)	0.29 (2.055)	0.000
When you are shopping	2.53 (0.950)	1.28 (1.785)	0.000
When you are watching TV	2.55 (0.964)	1.54 (1.630)	0.000
When you are doing homework	2.57 (0.919)	1.71 (1.446)	0.000
When you are on your way from school	2.52 (1.017)	0.64 (2.126)	0.000
**Intention**			
Own intention to smoke in the next year	-1.89 (1.628)	0.20 (2.019)	0.000

* Mann-Whitney U test.

### Factors associated with smoking from logistic regression analysis

The results of logistic regression analyses are shown in Table 5, and they reveal that ownership of a family phone and a computer is associated with a lower risk of smoking behavior. In the second model were added risk behaviors (alcohol consumption, destroying other people’s things, fighting at least once a month, stealing at least once a month, spending frequently time in bars/discos/parties, school achievement); alcohol consumption at least once a month, being in the bottom two-thirds of the class, spending less of free time at home or most of free time in parks were associated with higher risk of smoking. In the third model, attitudes, social norms, social pressure, social modeling (smoking behavior of parents, siblings, best friend), and self-efficacy were added. Pro-smoking attitudes and social pressure were associated with higher odds of smoking, while higher levels of self-efficacy were associated with lower risk of smoking behavior. Also, the best friend’s smoking behavior was associated with higher odds of being a smoker. In the fourth model, the intention to smoke in the next year was added, and the final model shows that smoking was associated with higher intention to smoke in the next year, stronger social pressure, and attitudes favorable to smoking and lower self-efficacy in refraining from smoking in different situations. Lower academic performance and more frequent consumption of alcohol also increased the risk of smoking.

**Table 5:** Binary logistic regression for the associations with smoking behaviors, Arad and Cluj Counties, Romania, 2019 (N=748)

*Variables*	*Model 1* *OR (95% CI)*	*Model 2* *OR (95% CI)*	*Model 3* *OR (95% CI)*	*Model 4* *OR (95% CI)*
**Household phone**	0.375** (0.218–0.644)	0.348*** (0.192–0.631)	-	-
**Household computer**	0.521* (0.303–0.895)	-	-	-
**Using alcohol at least once a month**		4.266*** (2.038–8.929)	5.134** (2.033–12.963)	3.746** (1.421–9.873)
**School achievement**		0.320*** (0.170–0.602)	0.428* (0.204–0.899)	0.380* (0.176–0.818)
**Spending free time at home**		0.316** (0.153–0.649)	-	-
**Spending free time in the park**		1.740* (1.006–3.011)	-	-
**Pro-smoking attitude**			1.069* (1.012–1.128)	1.064* (1.007–1.124)
**Social pressure**			1.122** (1.038–1.213)	1.106* (1.021–1.198)
**Social modeling - best friend**			2.195* (1.090–4.418)	-
**Self-efficacy**			0.943*** (0.925–0.962)	0.957*** (0.937–0.977)
**Intention to smoke in the next year**				1.382** (1.136–1.681)

The dependent variable was smoking behavior. The independent variables included in the analysis in Model 1 were sociodemographic variables [gender, living with both parents, ownership of a personal mobile phone, family ownership of a private property (apartment/house), phone, mobile phone, car, computer, TV]. In Model 2, risk behavior (use of alcohol at least once a month, gambling, fighting, destroying/stealing other people’s things at least once a month), school achievement, and the most frequented places in their free time (at home, friend`s home, bars/discos, parks, practicing sports) were included. In Model 3 were included the scales regarding attitudes, social norms, social pressure, social modeling, and self-efficacy items, while intention to smoke in the next year was included in Model 4.

* p<0.05.

** p<0.01.

*** p<0.001.

## DISCUSSION

This study focused on the smoking behavior of Romanian school students, aged 13–14 years, from rural areas. Previous studies performed among Romanian junior high school students and senior high school students observed a higher smoking rate among boys than girls, but our study showed a prevalence of 15.9% of current smokers without noticing gender differences^17^.

Various studies paid attention to the connection between socio-economic level and smoking habits, showing that there are differences based on region, country, area, and year when the study was performed^18,19^. A study performed in 2004 among Romanian high school students from an urban area revealed higher smoking rates among adolescents with many household items. During that time, Romania was considered a low-income country. Nowadays, Romania is considered a high-income country, and in our study, we found that smoking was more frequent among those with a lower socio-economic level, as indicated by having fewer household items^10^.

On the other hand, our findings align with previous research from 2014 among 7th and 8th grade students from other three counties in Romania, indicating a correlation between low academic achievement and tobacco use among adolescents^20^.

Our research revealed a significant association between smoking and various risky behaviors like vandalism, frequent alcohol use, gambling, and participation in physical altercations. Other studies performed among similar populations observed a correlation between smoking and other risky behaviors, especially alcohol consumption, which significantly increases the likelihood of smoking^13,21-23^.

In alignment with other studies, our research found that adolescents engaging in sports activities were less likely to smoke^22^ On the other hand, our study showed greater smoking odds among adolescents who frequented bars and discos, similar to a Romanian study among high school students from urban areas^10^.

Regarding attitudes, our study found that smokers were more likely to believe that smoking offers benefits such as feeling more attractive, losing weight, gaining confidence, and achieving better social integration. Non-smokers were more likely to identify the bad side of smoking, such as being unhealthy, wrong, and unfriendly, as in other similar studies among adolescents^10,24^.

Similar to other studies, the results of our study revealed that social norms, pressure, and smoking behavior of family members play a significant role in influencing smoking behavior among school students^10,18-20^. With regard to smoking behavior of family members, we noticed that there were no differences between smokers and non-smokers school students with regard to smoking behavior of father, but the smoking was more frequent among mothers and siblings of smokers; a study performed several years ago among Romanian school students from urban areas also found this and it might be the fact that smoking is more frequent among men than women among adults in Romania, but when mothers also smoke this increases the risk of smoking of children^10^. Peer influences were also significant, adolescent smoking behavior being tied to the actions, norms, and pressure of friends and peers in the same academic year, as other studies also underlined^25-30^.

Our study found that non-smokers had greater confidence in their ability to resist smoking across different situations, while smokers reported lower self-efficacy in avoiding smoking in different social, contextual, and emotional situations. Research reveals that high self-efficacy is associated with lower smoking intentions in social, contextual, and emotional situations, while lower self-efficacy is linked to higher odds of smoking^10,31^. A longitudinal study conducted among junior high school non-smoking students in Romania also showed that smoking behavior was related to this outcome by showing that lower self-efficacy and high peer pressure significantly increased the risk of non-smokers to become smokers nine months later^12^. As expected, smokers have declared a higher intention to smoke in the future than non-smokers.

The results of the logistic regression analysis showed that motivational factors with the strongest association with smoking behavior were: pro-smoking attitudes, higher social pressure, and lower self-efficacy, as well as stronger intention to smoke in the next year. At the same time, alcohol consumption and lower school achievement increased the risk of smoking behavior.

Our results indicate that smoking prevention activities should strengthen adolescents’ attitudes toward the benefits of not smoking, expose the inaccuracies of some perceived advantages of smoking, and highlight the tendency of smokers to downplay the disadvantages of smoking. Prevention programs containing structured information regarding the disadvantages of smoking and debunking myths regarding smoking (e.g. it helps to be slim, it helps to calm the nerves, or to have a better social integration) can be useful for adolescents. Also, prevention programs should help school students to identify direct and indirect pressure to smoke and develop refusal skills in different situations and to improve the self-efficacy of school students to remain non-smokers.

A school-based peer-led smoking prevention program (‘I do not smoke’) based on these factors has been previously implemented and evaluated among Romanian junior high-school students from urban areas. The results of the program showed that those who participated in the program had a more negative attitude towards smoking, increased self-efficacy levels, and a more negative intention towards smoking, and also had a significantly lower risk of becoming smokers at follow-up^32^. This program might be used as a model for developing smoking prevention programs for rural adolescents in Romania. Future studies should pay attention to approaches for adapting and finding appropriate mechanisms for the implementation of the ‘I do not smoke’ program in rural areas, paying attention to the characteristics of adolescents from these areas, but also to barriers and facilitators for the implementation of such activities.

Given the fact that several adolescents are already smokers, smoking cessation messages and interventions should be developed to educate and motivate them with regard to the importance of making action plans for quitting^33,34^. Our research showed that smokers’ first intention when they want to quit is to inform their friends. On the other hand, fixing a date to quit smoking is the last endorsed quitting strategy in our study, even though it is very important in making a quit attempt. This can be an actionable target for intervention among adolescents by creating educational campaigns and smoking cessation programs that underscore the benefits of setting a quit date and provide practical tools and support to help participants commit to this step.

### Limitations

This study has several limitations. Firstly, the study only included middle school students from villages in two counties of Romania, limiting the generalizability of the results to urban settings or other regions of the country. Also, the data are based on self-reports of the participant, which can cause recall bias or social desirability bias; the reports on parents, siblings, and friends smoking were based on the middle school students’ own perception, which can also cause biases. Data were collected several years ago, which can be a limitation considering potential changes in adolescents’ habits and influences during this time. The cross-sectional design of the study captures data at a single point, limiting the possibility of inferring causality and observing changes over time. Also, obtaining consent from the parents in rural communities could introduce selection bias due to the fact that some of them did not agree with their child’s participation in the study.

## CONCLUSIONS

This study is one of the few assessing smoking behavior conducted among adolescents from rural Romania, and the first focusing on identifying factors associated with smoking and describing the differences between smokers and non-smokers based on the I-Change Model in rural areas of the country.

Although this study was conducted before the COVID-19 pandemic, its findings remain highly applicable for the development of smoking prevention programs in schools during the post-pandemic era. The pandemic has underscored the importance of public health initiatives, especially in addressing respiratory health and smoking as a risk factor. Our research data can provide valuable guidance for designing and implementing effective prevention strategies in schools, addressing both pre-existing and emerging challenges to ensure that students have the attitudes, skills, and support to make healthy choices.

## Supplementary Material



## Data Availability

The data supporting this research are available from the authors on reasonable request.
