# Supplementary material for: Smoking habits and factors that influence them among school students from rural areas of Romania
Source: Tob Induc Dis. 2026 Feb 14;24:10.18332/tid/214406. doi: 10.18332/tid/214406 (PMC12906254; doi:10.18332/tid/214406)
Supplement: Supplementary file 1 [file TID-24-19-s1.pdf]

Supplementary Table 1.

Table 1. Plan for quitting smoking among smokers, Romania, 2019 (N=119)

	Percentage (%)
Inform friends	
Totally disagree/disagree	7.6
I don`t know	26.9
Totally agree/agree	65.5
Hide everything correlates to smoking	
Totally disagree/disagree	13.5
I don`t know	29.4
Totally agree/agree	57.1
Promise myself a reward	
Totally disagree/disagree	15.9
I don`t know	34.5
Totally agree/agree	49.6
Simply stop	
Totally disagree/disagree	15.9
I don`t know	31.1
Totally agree/agree	52.9
Convince someone to join me	
Totally disagree/disagree	11.7
I don`t know	34.4
Totally agree/agree	48.7
Would prepare to resist temptation	
Totally disagree/disagree	11.7

I don`t know<br>Totally agree/agree	28.6<br>59.7
Fix a date to quit smoking<br>Totally disagree/disagree<br>I don`t know<br>Totally agree/agree	 24.4<br>40.3<br>35.3
Read brochures about quitting smoking<br>Totally disagree/disagree<br>I don`t know<br>Totally agree	 14.2<br>32.8<br>53

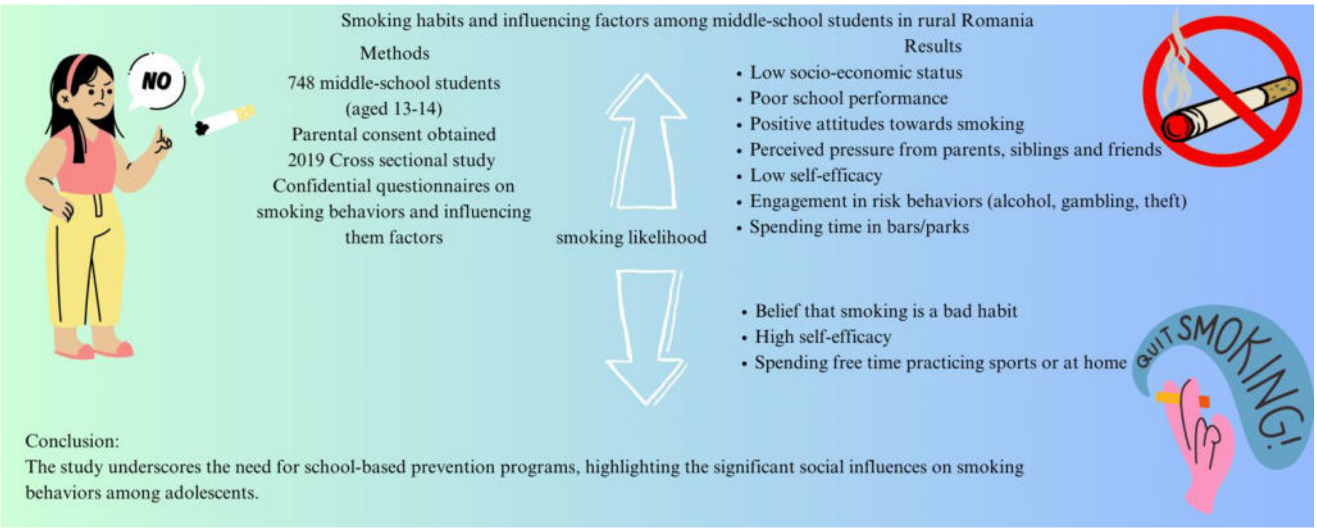

© 2026 Tudor T.E. et al.
